# Metabolic dysfunctions in multiple sclerosis: implications as to causation, early detection, and treatment, a case control study

**DOI:** 10.1186/s12883-015-0411-4

**Published:** 2015-08-27

**Authors:** Vijitha K. Senanayake, Wei Jin, Asuka Mochizuki, Bassirou Chitou, Dayan B. Goodenowe

**Affiliations:** Phenomenome Discoveries Inc, 204-407 Downey Road, Saskatoon, SK S7N 4L8 Canada

## Abstract

**Background:**

Biochemical changes associated with multiple sclerosis (MS), and its various clinical forms have not been characterized well. Therefore, we investigated the biochemistry of MS in relation to its natural history using targeted lipidomics platforms.

**Methods:**

Cross-sectional serum samples from 24 secondary progressive (SPMS), 100 relapsing remitting (RRMS), 19 primary progressive MS (PPMS), and 55 age-matched control subjects were analyzed by flow injection tandem mass spectrometry for very long chain fatty acid (VLCFA) containing phosphatidyl ethanolamines (PtdEtn), plasmalogen ethanolamines (PlsEtn) and for novel anti-inflammatory gastrointestinal tract acids (GTAs). Changes in analyte levels relative to healthy controls were correlated with the disease stage and disease duration.

**Results:**

RRMS subjects having <13 years disease duration had elevated levels (*p* < 0.05) of anti-inflammatory metabolites (GTAs) and normal levels (*p* > 0.05) of mitochondrial stress biomarkers (VLCFA-PtdEtn), compared to controls. SPMS subjects had statistically similar levels of anti-inflammatory metabolites (GTAs), elevated mitochondrial stress metabolites (VLCFA-PtdEtn) and elevated peroxisomal metabolites (PlsEtn) compared to controls (*p* < 0.05). RRMS subjects with > = 13 years disease duration exhibited metabolic profiles intermediate between short-duration RRMS and SPMS, based on statistical significance. Therefore, RRMS cohort appear to comprise of two metabolically distinct subpopulations. The key clinical discriminator of these two groups was disease duration. PPMS patients exhibited metabolic profiles distinct from RRMS and SPMS.

**Conclusions:**

These data indicate that inflammation and mitochondrial stress are intricately involved in the etiology of MS and that progression in MS can potentially be monitored using serum metabolic biomarkers.

## Background

Multiple sclerosis (MS) is a central nervous system disease that progresses to severe disability. The relatively young age of onset of the disease and the progressive debilitation place a severe burden on national health systems and the families of the affected person.

MS manifests in several forms. Clinically Isolated Syndrome (CIS) is the first manifestation of MS-like signs and symptoms, usually followed by another attack at which a clinical diagnosis of MS is made. Most patients are initially diagnosed with relapsing remitting MS (RRMS). The RRMS form is characterized by sudden relapses punctuated by short or long-term remissions. Eighty percent of RRMS patients eventually progress to secondary progressive MS (SPMS), which has a progressive course resulting in severe, irreversible debilitation [1]. Primary progressive MS (PPMS) is a progressive type of MS without an initial relapsing and remitting period.

The diagnosis of MS is a daunting and lengthy task as definitive diagnostic tests are not available. The physician has to rely on the clinical presentation and Magnetic Resonance Imaging (MRI) evidence of the appearance of lesions separated in time and space for diagnosis [2–4]. Other manifestations of the disease (e.g., optic-spinal form) and diseases with similar symptoms make MS diagnosis challenging [4–6]. Phadke and Best [5] stated that the only proven diagnosis of MS is through autopsy, highlighting the difficulty in diagnosing MS. Therefore, the need for effective and definitive diagnostic tests for MS cannot be overemphasized.

The biggest hindrance for the development of a diagnostic or predictive test for MS is the lack of complete understanding of its causes and disease progression process. The pathological process in MS is a dynamic process involving changes in cellular turnover and metabolism. Accordingly, a novel concept in disease risk prediction and diagnosis is monitoring the metabolic trail elicited by these changes. We have previously reported on the discovery of a novel class of anti-inflammatory hydroxylated long chain fatty acids called Gastro-intestinal Tract Acids (GTAs) present in human serum [7]. These metabolites were decreased in diseases such as colorectal cancer and inflammatory bowel disease [8]. In addition, we have also reported mitochondrial stress induced changes in the levels of ethanolamine phospholipids [9] in autism, a disease characterized by central nervous system (CNS) inflammation [9]. Since MS is a progressive disorder with a long disease duration, and given that inflammation [10, 11] and oxidative stress-induced mitochondrial dysfunction [12–14] are implicated in MS, metabolic signatures of these events could possibly be used to monitor the underlying disease processes and could be helpful in assessing response to treatment. Hence the objective of this study is to investigate the biochemical changes associated with MS and its clinical forms in relation to the natural history of the disease.

## Methods

### Study subjects

A cohort of MS patients representing the three forms of the disease and age matched controls was selected for the study. Serum samples were collected, processed and stored in a consistent manner using standardized protocols and operating procedures at BioServe Biotechnologies, Inc., Laurel, Maryland, USA (formerly Genomics Collaborative Inc., a subsidiary of SeraCare Life Sciences Inc.). Collection protocols were approved by the Western Institutional Review Board, and all samples were properly consented. The research did not contravene the principles of the Declaration of Helsinki (http://www.wma.net/en/30publications/10policies/b3/index.html). Detailed clinical and diagnostic data were obtained from the suppliers of serum samples. The number of patients with RRMS, PPMS, and SPMS were 100, 19 and 24 respectively. Control samples were from 55 healthy subjects.

### Lipidomic analyses

Three metabolite classes, namely phosphatidylethanolamines (PtdEtn), plasmalogen ethanolamines (PlsEtn), and a unique class of hydroxylated unsaturated very long chain fatty acids called Gastrointestinal Tract Acids (GTAs) were examined. Serum samples were stored at −80 °C until analysis. Sample extraction and analysis was carried out essentially as described earlier [15, 9], utilizing flow injection tandem mass spectrometry and monitored under negative atmospheric pressure chemical ionization mode (APCI). The method was based on multiple reaction monitoring (MRM) of parent/fragment ion transitions specific for each metabolite. All samples were analyzed in a randomized blinded manner. Results were based on ratios of integrated analyte peak area to the internal standard (PtdEtn and PlsEtn) or based on the calculated concentration extrapolated from an external standard curve (GTA).

### Statistical analyses

Log transformed relative intensities of each analyte was used for statistical analyses. Analysis of variance (ANOVA) with post hoc Sidak test or student’s *t*-test was used for statistical analysis. For both ANOVA and student’s *t*-test, *p* < 0.05 was considered significant. All computations were done using STATA version 13 (Stata, RRID:nlx_156918) or Microsoft Excel, 2010.

## Results

### Patient characteristics

Mean ages in years (95 % CI) of controls, RRMS, SPMS, and PPMS cohorts were 48.3 (45.7–50.8), 46.6 (44.7–48.5), 51.3 (47.2–55.4), and 54.7 (50.6–58.8), respectively. Percentages of males and females in control, RRMS, SPMS and PPMS groups in that order were 22 and 78 %, 10 and 90 %, 33 and 67 %, and 42 and 58 %, respectively. Mean disease durations (95 % CI) in RRMS, SPMS, and PPMS patients were 9.6 (7.9–11.3), 11.9 (9.4–14.4) and 12.7 (7.9–17.6), respectively.

### Ethanolamine phospholipids

**Fig. 1:**
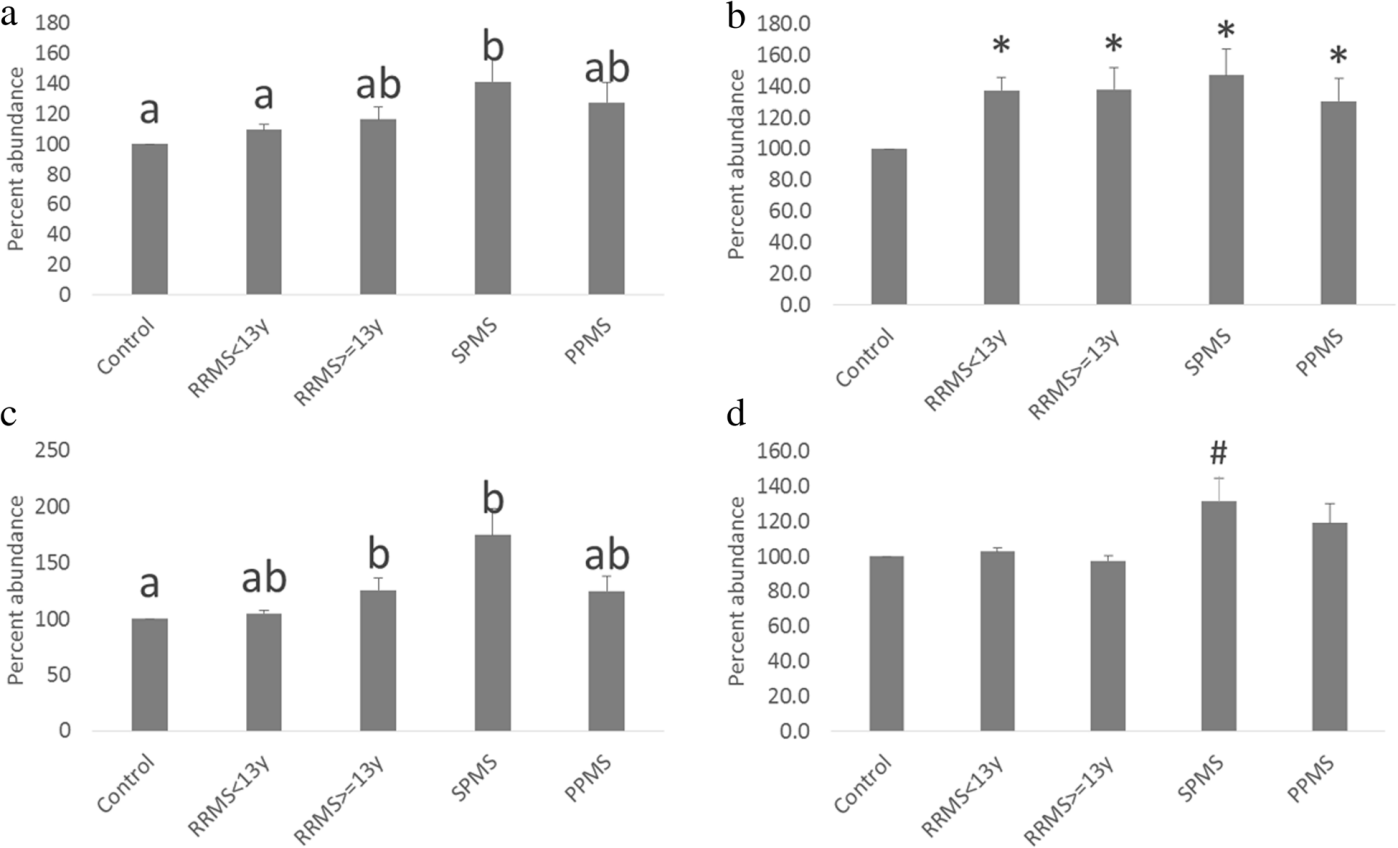
**a**. Phosphatidyl ethanolamine 16:0/28:0 levels in Multiple Sclerosis. Cross sectional serum samples from each of the clinical groups were subjected to lipidomic analysis utilizing tandem mass spectrometry. Bars represent mean value of the ratio of phosphatidyl ethanolamine 16:0/28:0 to the internal standard. Error bars represent ± SEM. RRMS <13 Y: Relapsing Remitting Multiple Sclerosis having less than 13 years of disease duration; RRMS > = 13 Y: Relapsing Remitting Multiple Sclerosis with >= 13 years of disease duration; SPMS: Secondary Progressive Multiple Sclerosis; PPMS: Primary Progressive Multiple Sclerosis. Letters shared in common indicate no significant difference between the respective groups ( *p* < 0.05, ANOVA followed by post hoc Sidak test of log normalized internal standard corrected analyte values). **b**. Ratio of Phosphatidyl ethanolamine 16:0/22:6 to phosphatidyl ethanolamine 16:0/18:3 in Multiple Sclerosis. Cross sectional serum samples from each of the clinical groups were subjected to lipidomic analysis utilizing tandem mass spectrometry. Bars represent mean value of phosphatidyl ethanolamine 16:0/22:6 normalized to phosphatidyl ethanolamine 16:0/18:3. Error bars represent ± SEM. RRMS <13 Y: Relapsing Remitting Multiple Sclerosis having less than 13 years of disease duration; RRMS > = 13 Y: Relapsing Remitting Multiple Sclerosis with >= 13 years of disease duration; SPMS: Secondary Progressive Multiple Sclerosis; PPMS: Primary Progressive Multiple Sclerosis. * *p*  < 0.05 vs controls (Student’s test). **c**. Plasmalogen ethanolamine 16:0/22:6 levels in Multiple Sclerosis. Cross sectional serum samples from each of the clinical groups were subjected to lipidomic analysis utilizing tandem mass spectrometry. Bars represent mean value of the ratio of plasmalogen ethanolamine 16:0/22:6 to the internal standard. Error bars represent ± SEM. RRMS <13 Y: Relapsing Remitting Multiple Sclerosis having less than 13 years of disease duration; RRMS > = 13 Y: Relapsing Remitting Multiple Sclerosis with >= 13 years of disease duration; SPMS: Secondary Progressive Multiple Sclerosis; PPMS: Primary Progressive Multiple Sclerosis. Letters shared in common indicate no significant difference between the respective groups ( *p* < 0.05, ANOVA followed by post hoc Sidak test of log normalized internal standard corrected analyte values). **d**. Ratio of Plasmalogen ethanolamine 16:0/22:6 to phosphatidyl ethanolamine 16:0/18:3 in Multiple Sclerosis. Cross sectional serum samples from each of the clinical groups were subjected to lipidomic analysis utilizing tandem mass spectrometry. Bars represent mean value of plasmalogen ethanolamine 16:0/22:6 normalized to phosphatidyl ethanolamine 16:0/18:3. Error bars represent ± SEM. RRMS <13 Y: Relapsing Remitting Multiple Sclerosis having less than 13 years of disease duration; RRMS > = 13 Y: Relapsing Remitting Multiple Sclerosis with >= 13 years of disease duration; SPMS: Secondary Progressive Multiple Sclerosis; PPMS: Primary Progressive Multiple Sclerosis. # *p* = 0.09 vs controls (Student’s test)

Table 1 summarizes the observed changes in PtdEtn species according to MS disease state and duration. Most of the PtdEtn containing very long chain fatty acids (VLCFA) assayed were elevated in SPMS vs controls (*p* < 0.05) (Table 1a). In comparison to the controls, only 11 VLCFA species were significantly elevated in RRMS patients with a lesser disease duration (<13 y) (Table 1b). However, when disease duration in RRMS exceeded 13 y, the number of significantly elevated VLCFA species (vs controls) increased to 27 (Table 1c). The number of VLCFA species significantly elevated (vs controls) in PPMS patients were not as high as SPMS (Table 1d) and tended to be similar to the RRMS >13 y category (Table 1c and Table 1d). Significant elevation of 28:0 containing PtdEtn (PtdEtn 16:0/28:0), was observed in SPMS compared to the controls and RRMS < 13 y (Fig. 1a). When RRMS patients were divided into two groups based on disease duration, the group having the longer disease duration (> = 13 y) had levels not different from SPMS (*p* > 0.05) (Fig. 1a). In general, the level of circulating VLCFA in RRMS tended to increase with their disease duration (data not shown).

**Table 1:** Relative changes in fatty acid classes at sn-2 position of phosphatidyl ethanolamines in Multiple Sclerosis

a. SPMS patients compared to healthy controls												
Chain length→	18	20	22	24	26	28	30	32	34	36	38	40
No. of double bonds ↓												
0			16/18↑	16/18↑	16/18↑	16/18↑		16/18↑		16↑	16/18↑	16/18↑
1	18↑		16/18↑		18↑			18↑	16↑	16↑	16/18↑	16/18↑
2			16↑				16↑		16↑	16↑	16↑	
3	16/18↑	16/18↑	16/18↑	16/18↑	16/18↑	16/18↑	16/18↑	16↑	16↑	16↑	16/18↑	16/18↑
4		16/18↑	16/18↑	16/18↑	16/18↑	16/18↑	18↑	16↑	16↑	16/18↑	16/18↑	16/18↑
5		16/18↑	16/18↑	16/18↑	16/18↑	16↑	16↑	16↑	16↑	18↑	16/18↑	16/18↑
6		16/18↑	16/18↑	16/18↑	16/18↑	18↑	16↑				18↑	16↑
b. RRMS patients with a disease duration <13 years compared to healthy controls												
Chain length →	18	20	22	24	26	28	30	32	34	36	38	40
No. of double bonds ↓												
0			18↑		16↑							
1												
2												
3									16↑			
4								18↑				
5					16↑						16/18↑	16↑
6		18↑	16↑						16↑			
c. RRMS patients with a disease duration > =13 years compared to healthy controls												
Chain length →	18	20	22	24	26	28	30	32	34	36	38	40
No. of double bonds ↓												
0			18↑		16↑	16↑					16↑	
1						18↑				16↑		
2			16↑				16/18↑	18↑		16↑	16↑	
3		16↑	16↑				18↑		16↑	18↑	16↑	
4												
5					16↑						16↑	
6		16/18↑	16/18↑	16↑	16↑				16↑			
d. PPMS patients compared to healthy controls												
Chain length →	18	20	22	24	26	28	30	32	34	36	38	40
No. of double bonds ↓												
0			18↑	18↑	18↑	16/18↑				18↑	16/18↑	
1			18↑						18↑			
2								18↑				18↑
3					16↑		16↑		16↑			
4		18↑			16↑	16↑	18↑					
5												
6		16/18↑	16/18↑	16/18↑			16↑				18↑	

Fatty acids at sn-2 position in phosphatidyl ethanolamines are categorized according to their number of double bonds and chain length. Carbon chain length is given in columns and number of double bonds are given in rows. Fatty acid chain length at sn-1 position is given within the cells as 16 or 18, indicating 16:0 and 18:0 respectively. Up and down arrows indicate a significant increase or decrease in relative intensity (ratio to internal standard) respectively, compared to the control group at *p* < 0.05 (student’s t-test). a. SP-MS patients compared to healthy controls, b. RRMS patients with a disease duration <13 years compared to healthy controls, c. RRMS patients with a disease duration >=13 years compared to healthy controls, d. PPMS patients compared to healthy controls

The ratio of PtdEtn containing Docosahexaenoic acid (DHA, 22:6) to PtdEtn containing the precursor (18:3) was significantly enhanced in RRMS > = 13 y, SPMS and PPMS compared to controls (Fig. 1b). This enhancement was seen even in early stages (<13 y disease duration) of RRMS (Fig. 1b). DHA biosynthesis occurs via chain elongation and desaturation of 18:3 (n-3) to 24:6 in the endoplasmic reticulum followed by beta oxidation to 22:6 in the peroxisome [16]. These observations indicate that enhanced flux through this pathway occurs early in the disease process. Plasmalogen biosynthesis involves partial beta-oxidation of VLCFA to medium chain fatty acids prior to conversion to its alcohol followed by elongation to predominately 16 and 18 carbon alcohols that eventually get incorporated at the sn-1 position [17]. Because of the direct role played by peroxisomes in plasmalogen biosynthesis, plasmalogen levels can be used as markers of peroxisomal function and activity. All plasmalogen species measured were significantly elevated in SPMS compared to the controls. As can be seen in Fig. 1c, DHA containing plasmalogen (PlsEtn 16:0/22:6), a representative plasmalogen, was significantly elevated in SPMS and RRMS > = 13 y (vs controls). When the ratio of PlsEtn 16:0/22:6 to PtdEtn 16:0/18:3 was taken in order to normalize for dietary variations of 16:0/18:3, the ratio was elevated only in SPMS patients relative to controls, confirming the enhanced synthesis of PlsEtn 16:0/22:6 in SPMS (Fig. 1d).

### Gastrointestinal tract acids

**Fig. 2:**
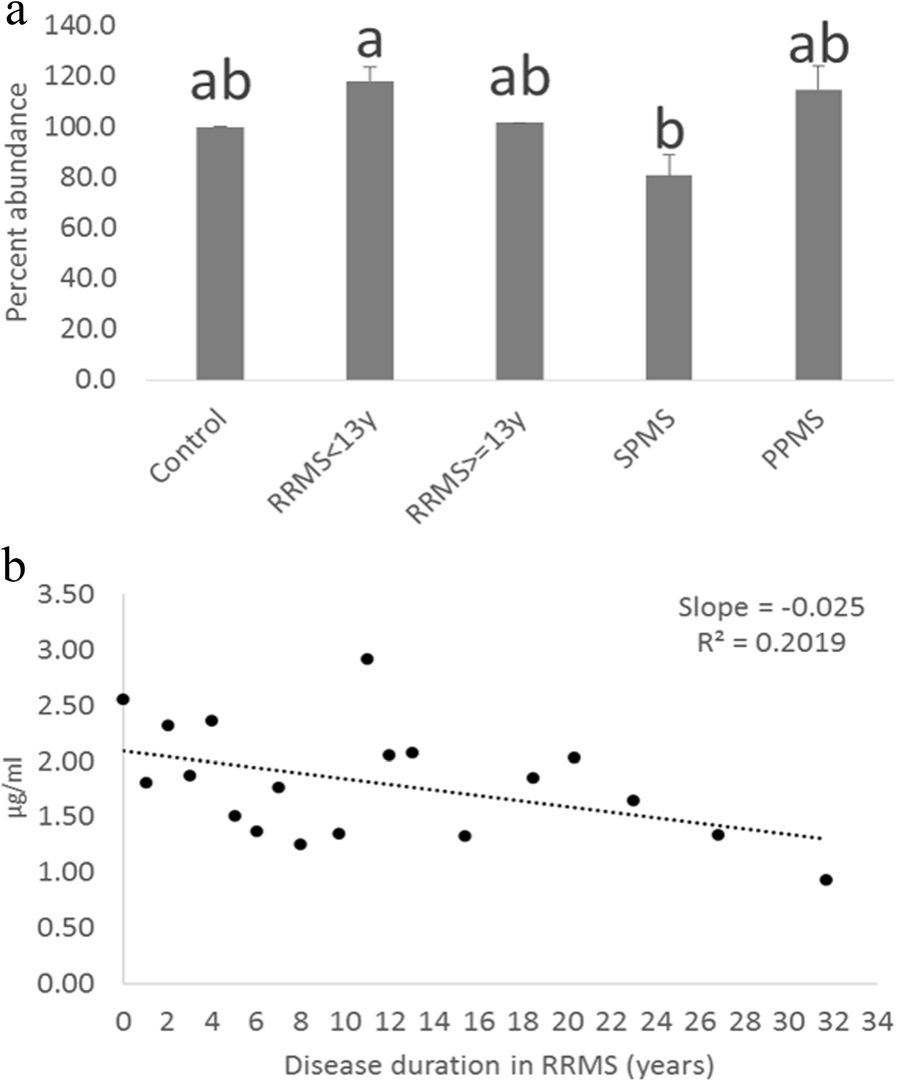
**a**. Gastrointestinal tract acid (465/403) levels in Multiple Sclerosis. Cross sectional serum samples from each of the clinical groups were subjected to lipidomic analysis utilizing tandem mass spectrometry. Bars represent the mean concentration of gastrointestinal tract acid (abbreviated as MRM 465/403). Error bars represent ± SEM. RRMS <13 Y: Relapsing Remitting Multiple Sclerosis having less than 13 years of disease duration; RRMS > = 13 Y: Relapsing Remitting Multiple Sclerosis with >= 13 years of disease duration; SPMS: Secondary Progressive Multiple Sclerosis; PPMS: Primary Progressive Multiple Sclerosis. Letters shared in common indicate no significant difference between the respective groups (*P* < 0.05, ANOVA followed by post hoc Sidak test of log normalized internal standard corrected analyte values). **b**. Correlation of gastrointestinal tract acid (465/403) to the disease duration in relapsing remitting Multiple Sclerosis. Mean concentration of gastrointestinal tract acid 465/403 in serum as measured by tandem mass spectrometry in relapsing remitting multiple sclerosis patients with the same disease duration (rounded down to years) were plotted against the disease duration. Slope of the curve that best fits the data was −0.025 and *R* ^*2*^ = 0.2019

Anti-inflammatory long-chain fatty acids (GTAs) were significantly elevated in short duration RRMS (disease duration <13 years) compared to SPMS (Fig. 2a). Table 2 shows statistically significant changes in levels of GTAs with different chain lengths and degree of unsaturation, in each clinical form, compared to controls. As shown in Table 2, significant elevation of 11 out of 34 GTAs analyzed were seen in RRMS patients with disease duration less than 13 years (vs controls). Eight out of 34 GTAs were significantly reduced in SPMS patients while one GTA was elevated in PPMS (vs controls). These trends are illustrated in Fig. 2a for a selected GTA (GTA465/403). SPMS patients demonstrated significant decrease of this GTA compared to RRMS < 13 y, while there was no significant difference between  > = 13 y and SPMS or between  > = 13 y and PPMS. Effect of disease duration was further illustrated by the negative correlation observed between disease duration and the level of GTA465/403 in RRMS patients (Fig. 2b).

**Table 2:** Relative changes in gastrointestinal tract acids in Multiple Sclerosis

Carbon length Number of → double bonds/free hydroxyl groups 1 ↓	C28	C30	C32	C34	C36
1/0	↑RR < 13y				
1/1	↑RR < 13y				↑RR < 13y
1/2	↓SP	↑RR < 13y	↑RR < 13y		
2/0	↑RR < 13y	↑RR < 13y			
2/1	↑RR < 13y,↓SP	↓SP	↑RR < 13y	↓SP	
3/0	↑RR < 13y, ↑PP				
3/1	↓SP		↓SP		
3/2			↑RR < 13y		↓SP
4/0					
4/1		↓SP			

Gastrointestinal tract acids are categorized according to their chain length, number of double bonds and the number of free hydroxyl groups. Carbon chain length is given in columns and the number of double bonds and free hydroxyl groups are given in rows, separated by a slash. Up or down arrows indicate a significant increase or decrease in serum concentration respectively, compared to the control group at *p* < 0.05 (Student’s t- test). RR < 13y; Relapsing remitting Multiple Sclerosis patients with a disease duration <13 years, RR > 13y; Relapsing remitting Multiple Sclerosis patients with a disease duration > =13 years, SP; Secondary progressive Multiple Sclerosis patients, PP; Primary progressive Multiple Sclerosis patients

### Correlation of the ratio of VLCFA and GTA to disease duration in RRMS

Since there was a reciprocal trend in VLCFA and GTA levels in RRMS patients in relation to disease duration, the ratio of these two biomarkers was examined to see whether there was a time-dependent pattern that can indicate any transitional stage in the biochemical state of RRMS patients. When the disease duration was plotted against the ratio of PtdEtn 16:0/28:0 to GTA465/403, it tended to decrease up to approximately 13 years and then tended to increase with the remaining disease duration (data not shown). Moreover, there was a strong negative correlation between PtdEtn 16:0/28:0 and GTA 465/403 in the first year of RRMS diagnosis (*r*^*2*^ = 0.47).

## Discussion

Primary findings in this study are three-fold. Firstly, significant elevations of multiple species of VLCFA-PtdEtn were observed in SPMS patients, relative to age-matched healthy controls. Similar increases were observed in RRMS patients also, but a comparatively lesser number of VLCFA-PtdEtns were involved. However, RRMS patients with longer disease duration (>13 y) had more VLCFA-PtdEtn species elevated than RRMS patients with lesser disease duration (<13 y). Secondly, PlsEtn, especially those containing DHA, were significantly increased in SPMS compared to controls. PlsEtn levels did not differ in RRMS relative to controls, irrespective of the disease duration. Thirdly, anti-inflammatory molecules (GTAs) were significantly reduced in SPMS relative to the controls. In contrast, RRMS patients had significantly elevated levels of these molecules, compared to controls and levels tended to decrease with advancing disease duration.

Our study population included a cohort of RRMS, SPMS, and PPMS patients varying in disease duration and age. The control group consisted of age and gender matched individuals with no known morbidity at the time of blood collection. Therefore, metabolic differences observed in MS clinical entities compared to the controls represent underlying metabolic abnormalities. Although the study is cross sectional, which is a limitation, study subjects represented disease duration varying from 0–34 years in RRMS and from 2–26 years in SPMS, enabling us to correlate the metabolic profiles with the natural history of the disease.

VLCFA are fatty acids having carbon chains longer than 22 carbons. Circulating levels of these fatty acids are elevated in a number of disorders, for example in X-linked adrenoleukodystrophy (X-ALD) [18, 19] and Zellweger spectrum disorders [20, 21]. ATP-binding cassette sub-family D member 1 (adrenoleukodystrophy protein), a very long chain acyl-CoA transporter in the peroxisomal membrane, is deficient in X-ALD [19], whereas in Zellweger spectrum disorders, multiple peroxisomal proteins are deficient [22]. PlsEtn, which are produced solely by the peroxisomes, are characteristically reduced in all these diseases [23, 24] as a result of the defective peroxisomes. However, compared to controls, PlsEtn levels were not deficient either in RRMS or in SPMS patients in our study, implying that peroxisomal deficits, as found in the aforementioned diseases, are not the cause of VLCFA elevation in MS patients in our study.

Long chain fatty acids, such as palmitate (16:0) and oleic (18:1) are β-oxidized in mitochondria whereas VLCFA are β-oxidized in peroxisomes to medium chain fatty acids (MCFA) [25]. MCFA are then transported to mitochondria for further oxidation [25]. The acetyl-CoA units produced as a result of peroxisomal β-oxidation are either utilized for anabolic reactions within the peroxisomes such as for plasmalogen synthesis [24, 17], shuttled out to cytosol for chain elongation of fatty acids (the process of synthesizing VLCFA) [26], or become substrates for other synthetic reactions, for example cholesterol synthesis.

Significance of this peroxisomal-mitochondrial interaction in VLCFA metabolism is elaborated in following studies. If fatty acid trafficking to the mitochondria is blocked by inhibiting Carnitine Palmitoyl Transferase I (CPT I), the oxidation of long chain fatty acids (for example 16:0 or 18:0) in peroxisomes increases in isolated rat hepatocytes [27]. Diversion of polyunsaturated fatty acids to peroxisomes for metabolism has been shown when mitochondrial β-oxidation is inhibited in rats [28]. Furthermore, drug induced inhibition of mitochondrial function in rats enhanced peroxisomal β-oxidation gene expression [29]. However, since peroxisomal metabolism has an anabolic outcome, the acetyl-CoA produced is used for chain elongation of fatty acid [26]. Elongation of fatty acids is a major source of circulating VLCFA [30]. Therefore, elevation of circulating VLCFA is a consequence of mitochondrial β-oxidation deficiency.

We have previously shown that glutamate can dose dependently inhibit β-oxidation of palmitate [9]. Potential mechanisms included reduced processing of acetyl-CoA via the tricarboxylic acid (TCA) cycle due to the inhibition of citrate formation and/or changes in the NADH/NAD^+^ ratio due to efflux of aspartate [31]. Both mechanisms lead to inhibition of β-oxidation [9] increasing the accumulation of acyl-CoA in the cytoplasm. Carnitine is used both for the removal of excess acyl-CoA from mitochondria as well as for fatty acid transport into mitochondria, so the shifting of this equilibrium towards acetyl-carnitine could possibly reduce the availability of carnitine [32] for CPT I-dependent fatty acid transport across outer mitochondrial membrane, further reducing fatty acid oxidation. Cytosolic acetyl-CoA is also used for malonyl-CoA production [33, 34], which provides additional impetus to inhibit mitochondrial fatty acid oxidation [35].

VLCFA are high affinity ligands for Peroxisome Proliferator-Activated Receptor α (PPAR-α) [36]. Increased peroxisomal activity along with increased availability of substrate (acetyl-CoA produced during peroxisomal β-oxidation) will drive PlsEtn synthesis [9], elevating their levels. Although not observed in RRMS patients where VLCFA were not upregulated as much as in SPMS, elevated PlsEtn levels were seen in SPMS patients. Therefore, chronic elevation of certain VLCFA during the RRMS stage as shown in this study can be expected to lead to an eventual increase in PlsEtn in SPMS.

Moreover, DHA containing PtdEtn and PlsEtn were also elevated in SPMS patients, but not in RRMS patients. Conversion of 24:6 to 22:6 by β-oxidation, which is the final step in DHA synthesis, occurs in peroxisomes [37]. DHA synthesis is regulated by PlsEtn, as demonstrated by the reduction of DHA levels in PlsEtn deficient cells [38]. When PlsEtn was stimulated in these cells by adding a precursor, DHA synthesis also increased [38]. Therefore, upregulation of PlsEtn synthesis, along with global enhancement of fatty acid elongation, can be expected to trigger DHA synthesis, elevating its levels as seen in SPMS.

**Fig. 3:**
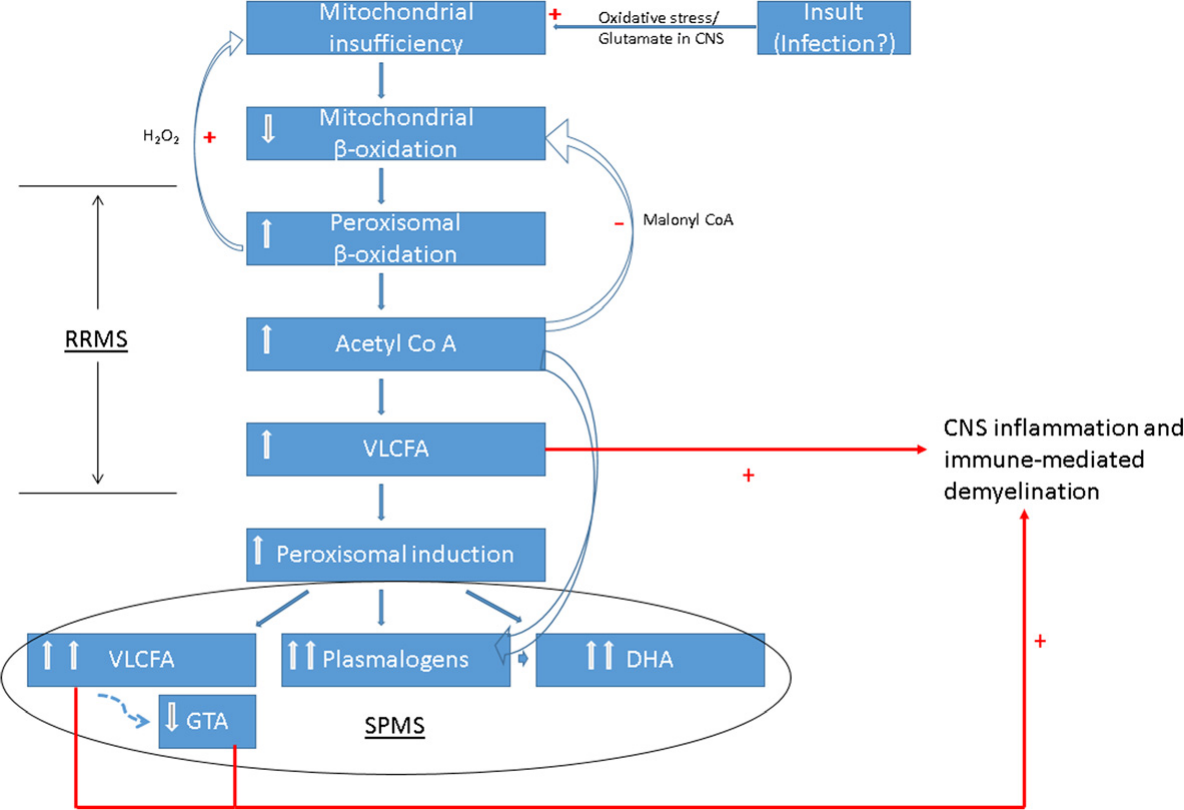
Schematic representation of the proposed relationship between Multiple Sclerosis, mitochondrial insufficiency and metabolic signatures. Underlying mitochondrial insuffciency possibly aggravated by an insult reduces mitochondrial capacity resulting in an increased flux through the peroxisomal β-oxidation system. This enhanced peroxisomal activity leads to elevated VLCFA and plasmalogens. Elevated VLCFA could result in CNS inflammation and demyelination. This metabolic derangement is hypothesized to start in early RRMS stage, culminating as a distinct metabolic phenotype in the SPMS stage. RRMS: Relapsing Remitting Multiple Sclerosis; SPMS: Secondary Progressive Multiple Sclerosis; VLCFA: Very Long Chain Fatty Acids; GTA: Gastrointestinal Tract Acids; DHA: Docosahexaenoic acid

Consequently, all lipid derangements observed in MS patients can be explained by the reduction of fatty acid β-oxidation due to subtle mitochondrial dysfunction (Fig. 3). Evidence supporting this hypothesis come from our previous *in vitro* studies using HepG2 cells that showed dose dependent reduction of palmitate oxidation by glutamate [9]. These cells also had increased levels of VLCFA and DHA containing PlsEtn [9]. Interestingly, L-carnitine supplementation has been reported to ameliorate fatigue in MS patients [39, 40], lending further support to this hypothesis. Moreover, a patient presenting with a relapsing and remitting demyelinating disorder similar to multiple sclerosis was found to have defects in mitochondrial β-oxidation and accumulated acylcarnitines in blood [41]. The patient improved with the supplementation of L-carnitine and riboflavin in the diet along with a low-fat, high-carbohydrate diet [41]. These clinical examples also provide indirect evidence for a likely reduction of mitochondrial fatty acid β-oxidation in MS.

Altered mitochondrial structure, molecular and biochemical abnormalities, impaired Complex I activity and impaired bioenergetics has been demonstrated in MS patients, as reviewed by Morris and Berk *et al.* [42]. Their review provide ample evidence that mitochondrial dysfunction is a frequent finding in MS. Mitochondrial dysfunction could be secondary to oxidative damage of this organelle. Oxidized macromolecules have been demonstrated in active MS lesions and are associated with apoptotic oligodendrocytes and areas of neurodegeneration in MS, as reviewed by Haider [43]. Oxidative stress in mitochondria could be induced by reactive oxygen and nitrogen species from activated microglia [13, 43]. Oxidative stress in turn could also induce pro-inflammatory genes, initiating a self-perpetuating cycle [42]. Therefore, an initial inflammatory trigger or incipient mitochondrial dysfunction triggered by environmental factors could initiate this vicious cycle.

VLCFA are known to cause inflammation and demyelination [44]. Chronic elevated levels of VLCFA can induce inflammation in the brain [24, 45]. Indeed, VLCFA infusion has induced lypooxygenases in the brain in experimental animals [46]. Demyelination is a hallmark of X-ALD, wherein deleterious effects of abnormally high VLCFA levels are well established. Sphingolipids that contain VLCFA are a major component of myelin, along with cholesterol and phospholipids [47]. They also exist in outer plasma membrane mostly in lipid rafts, in conjunction with cholesterol [48]. Elevation of VLCFA can therefore be expected to change sphingolipid composition. Perturbations in sphingolipid metabolism are known in MS [49]. Consequently, it can be speculated that there is a link between VLCFA, sphingolipids, and inflammation in MS.

Potential sources of elevated VLCFA are the liver and CNS. The liver is a source of many circulating lipids. As described above, myelin in the CNS is enriched with sphingolipids containing VLCFA. Demyelination can therefore possibly cause a local elevation of VLCFA, although it is unclear whether peripheral levels would be elevated. Also, activated microglia that emerge during demyelination produce copious amounts of glutamate [50] which can induce mitochondrial dysfunction. Mitochondrial dysfunction can lead to VLCFA accumulation as described elsewhere, possibly initiating and perpetuating a vicious cycle.

All three clinical forms of MS in this study had higher PtdEtn 16:0/28:0 than controls (*p* < 0.05, student’s *t*-test). However, an increase in the number of elevated VLCFA-PtdEtn species in patients with longer disease duration in RRMS, and an even higher number of elevated species in SPMS, may indicate that deranged VLCFA metabolism is an accumulating deficit in MS. In contrast, the opposite behavior of GTAs in these two patient categories is thought to be due to changes in the anti-inflammatory response. GTAs are novel fatty acids known to have anti-inflammatory effects *in vitro* [7] and are associated with a protective phenotype in various malignant tumors [24]. Higher GTA levels (*p* < 0.05 vs. controls) in early RRMS (<13 y disease duration) and low GTA levels (*p* < 0.05 vs. controls) observed in SPMS is suggestive of a protective response in early relapsing remitting phase, that eventually diminish in the progressive phase. Although unconfirmed, reduction in GTAs in the latter stages of RRMS could be ascribed to pathophysiological inability to sustain this protective response.

The reversal of the ratio of PtdEtn 16:0/28:0 to GTA 465/403 at about 13 years of disease duration in the RRMS group (data not shown) indicates that RRMS is comprised of two metabolically distinct sub-groups with the disease duration as the key discriminator between the subgroups (<13 y and > = 13 y). This is further confirmed by the differences in the total number of elevated VLCFA-PtdEtn and by significant differences in the levels of PtdEtn 16:0/28:0 and in GTA 465/403, observed between these two sub-groups. Although highly speculative without a longitudinal study, the presence  of metabolically distinct subpopulations in RRMS, with the subgroup with a disease duration > = 13 y being closer to SPMS (in terms of sum of elevated VLCFA-PtdEtn, PtdEtn 16:0/28:0 and GTA 465/403), indicate that transition from RRMS to SPMS can be monitored using these metabolic changes.

Longitudinal studies will be needed to overcome the limitations posed by cross-sectional nature of the RRMS samples. Smaller sample sizes in SPMS and PPMS patient groups limits the generalization of the results. Larger and longitudinal studies are planned to overcome these limitations.

## Conclusions

In summary, we have demonstrated that MS pathotypes have distinct metabolic profiles, characterized by overt changes in VLCFA and PlsEtn metabolism, and changes in putative anti-inflammatory molecules, GTAs. Interaction between these metabolites may predict the course of the disease. Further research, particularly longitudinal studies, are needed to verify this hypothesis.
